# Liver abscess caused by fish bone perforation of stomach wall treated by laparoscopic surgery: a case report

**DOI:** 10.1186/s40792-019-0639-0

**Published:** 2019-05-15

**Authors:** Tomoaki Bekki, Nobuaki Fujikuni, Kazuaki Tanabe, Hironobu Amano, Toshio Noriyuki, Masahiro Nakahara

**Affiliations:** 1 Department of Surgery, Onomichi General Hospital, 1-10-23 Hirahara, Onomichi, Hiroshima Japan; 2 Department of Gastroenterological and Transplant Surgery, Graduate School of Biomedical and Health Sciences, Hiroshima University, Kasumi 1-2-3 Minami-ku, Hiroshima, Hiroshima Japan

**Keywords:** Liver abscess, Perforation of gastrointestinal tract, Fish bone, Laparoscopic surgery

## Abstract

**Background:**

Formation of a liver abscess due to gastrointestinal perforation by a foreign body is rare. In addition, there are few case reports on laparoscopic surgical treatment of a liver abscess caused by perforation of the gastrointestinal tract by a foreign body.

**Case presentation:**

A 51-year-old man visited our hospital because of fever and anorexia. There were no physical findings except for fever. He had no comorbidities or surgical history. Laboratory tests showed increased inflammatory marker and liver enzyme levels. Abdominal ultrasonography showed a hypoechoic lesion in the left lobe of the liver. Abdominal contrast-enhanced computed tomography revealed an air-containing abscess in the left side of the liver and a high-density linear object. We diagnosed a liver abscess secondary to stomach perforation by a foreign body. Emergency laparoscopic surgery identified a fish bone in the abscess that formed between the stomach and liver. We succeeded in removing the fish bone laparoscopically.

The patient was discharged without any postoperative complications on day 11.

**Conclusions:**

A liver abscess secondary to perforation of the gastrointestinal tract by a foreign body usually requires surgical treatment. Foreign body removal is important to prevent recurrence of liver abscess. In cases with the foreign body located at the liver margin, a laparoscopic approach to the abscess is very useful.

## Background

Gastrointestinal foreign bodies are often encountered in clinical practice. Gastrointestinal perforation by a foreign body can lead to severe infection and abscess formation.

The most common etiologies of liver abscess include (1) complications of cholangitis, (2) bloodstream dissemination via the portal vein and hepatic artery in systemic sepsis, (3) local spread from infected contiguous tissue, and (4) traumatic injuries [1, 2]. Liver abscesses secondary to foreign body ingestion are extremely rare [3, 4].

The mortality rate from liver abscess has declined substantially, but ranges from 11 to 31% [5]. A liver abscess is usually discovered during workup for an infection in the absence of specific symptoms. Early diagnosis and treatment are essential.

There are a few case reports on liver abscess treated with laparoscopy. This report presents a case of liver abscess secondary to gastric wall perforation by a fish bone, with successful laparoscopic surgical treatment.

## Case presentation

**Fig. 1:**
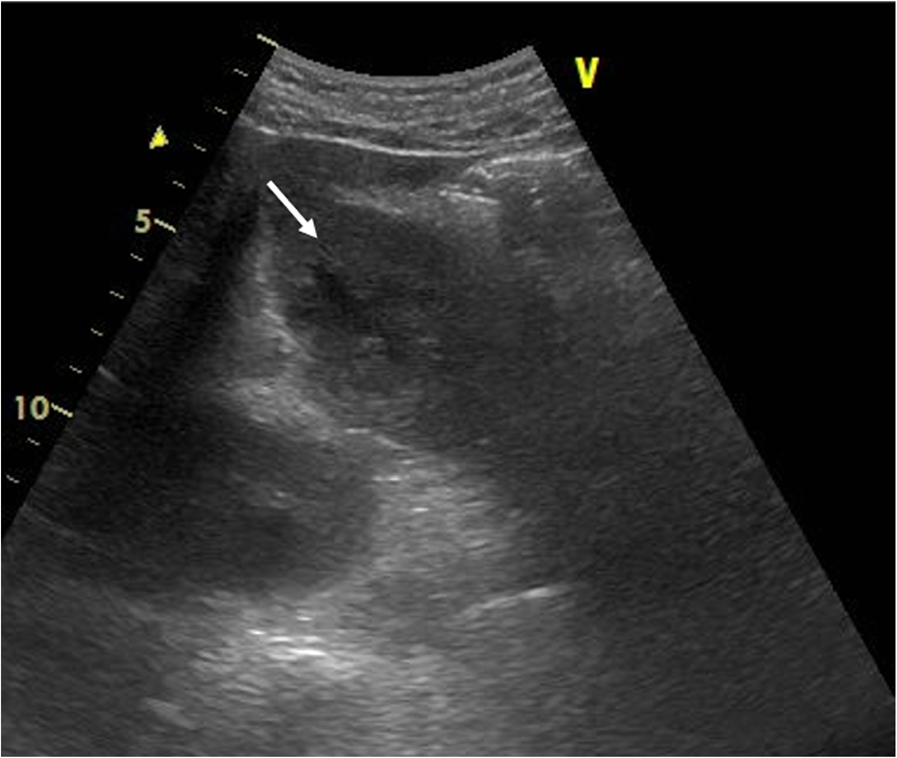
Abdominal ultrasonography findings. A hypoechoic lesion with an irregular margin (white arrow) was located in the left lobe of the liver

**Fig. 2:**
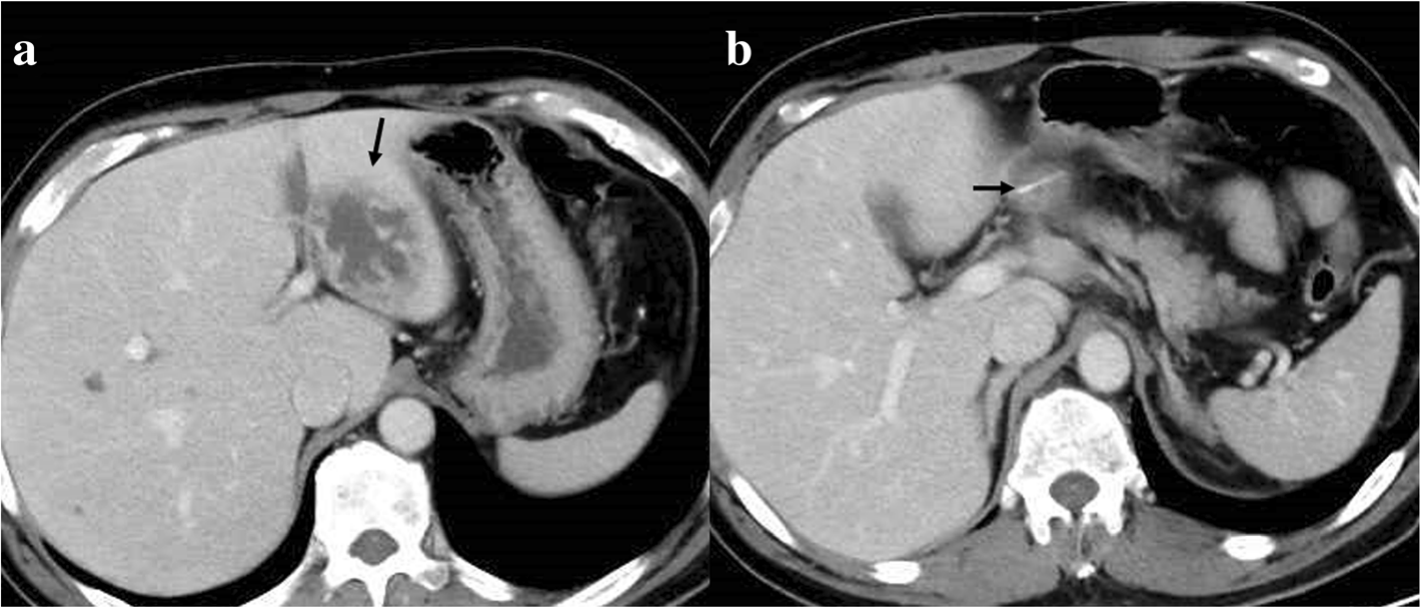
Abdominal contrast-enhanced computed tomography findings. **a** A lesion with coexisting low- and high-density areas (black arrow) was found in the left lobe of the liver. **b** A hyperdense linear body (black arrow) was found in the liver abscess.

**Fig. 3:**
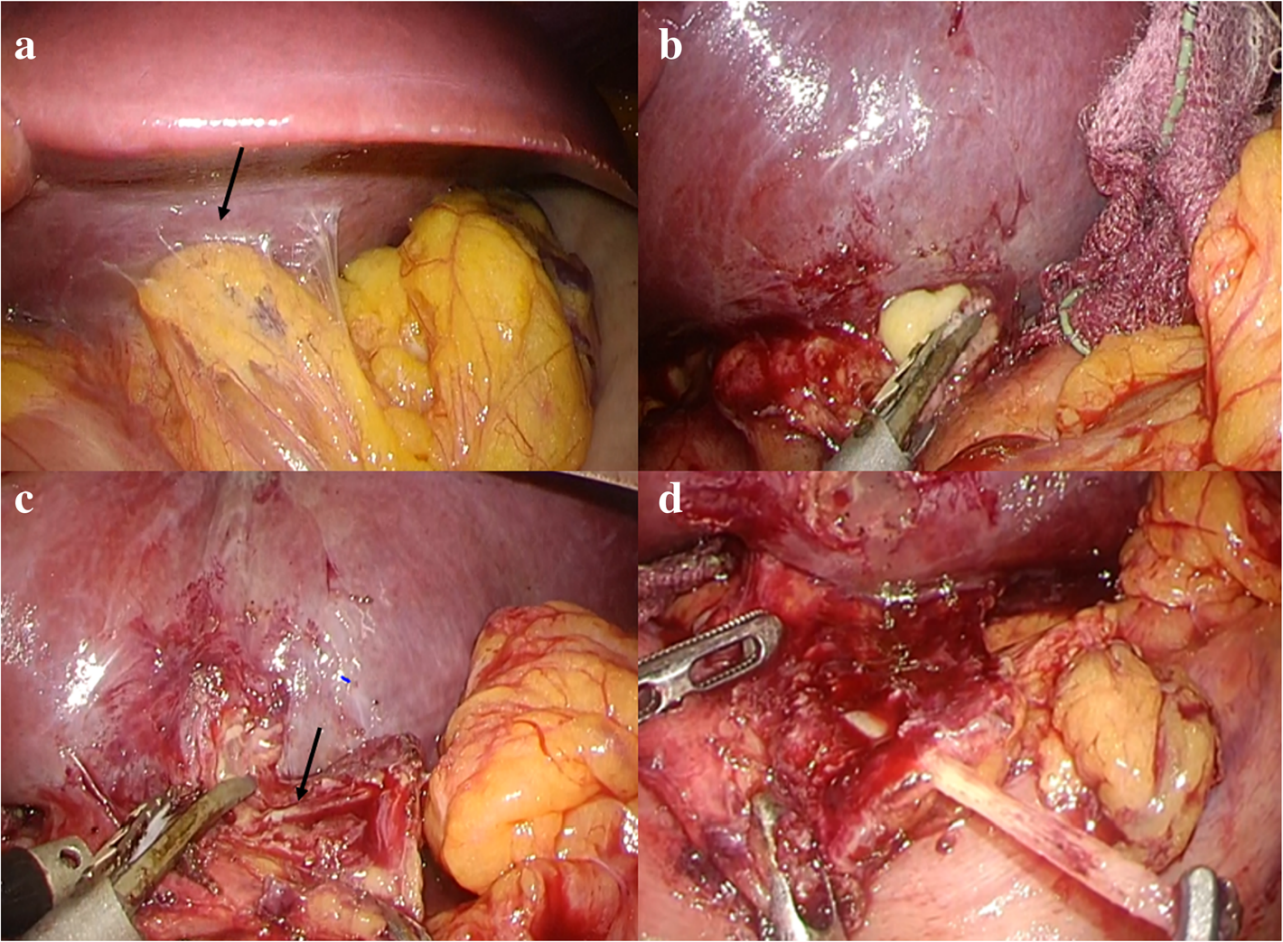
Intraoperative findings. **a** Adhesions (black arrow) formed between the liver and reticulum. **b** Pus leakage from the adhesions between the liver and reticulum. **c** Fish bone (black arrow) embedded in the reticulum. **d** Fish bone removed successfully with laparoscopic surgery

**Fig. 4:**
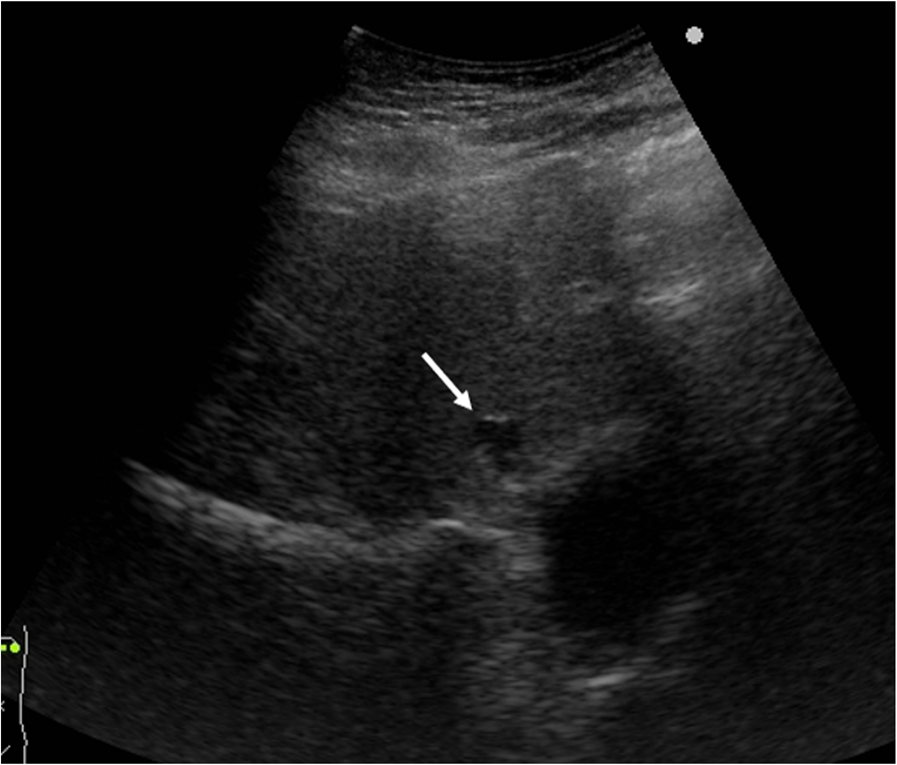
Postoperative abdominal ultrasonography findings. The abscess (white arrow) became smaller

A 51-year-old man was admitted to the Department of Surgery at our hospital for complaints of fever and anorexia. There was no abdominal pain, nausea, or abdominal distension. He had a high fever, anorexia, tachycardia, and tachypnea. He had no comorbidities or surgical history. The white blood cell count, liver enzymes, and C-reactive protein level were elevated. Abdominal ultrasonography showed a hypoechoic lesion with a maximum diameter of 40 mm in the left lobe of the liver (Fig. 1). Abdominal contrast-enhanced computed tomography (CT) revealed a lesion with coexisting low- and high-density areas in segment III of the liver. The lesion was adjacent to the stomach antrum and had a maximum diameter of 55 mm, with enhancement at the edge. The lesion contained air and a high-density linear object measuring about 24 mm (Fig. 2a, b). We suspected a liver abscess secondary to gastric perforation caused by a foreign body. The patient underwent abscess drainage and removal of the foreign body using five-port laparoscopic surgery. Adhesions had formed between the liver and reticulum due to inflammation (Fig. 3a). We confirmed pus leakage, performed lysis of adhesions, and found a fish bone inside the reticulum (Fig. 3b–d). The fish bone was removed laparoscopically. We lavaged the abscess cavity with saline. The operation was completed with the insertion of a drain inferior to the left lobe of the liver. There was no bile leakage from the abscess cavity. The total operative time was 62 min, and the total intraoperative blood loss was 20 ml. The pus culture showed the presence of *Streptococcus anginosus*, which matched the result of the blood culture. We used meropenem until postoperative day 10. The clinical course was uneventful, and the patient was discharged on postoperative day 11. When the patient was discharged, we changed the antibiotic treatment from meropenem to a combination of potassium clavulanate and amoxicillin hydrate according to the indications of the blood culture. On outpatient postoperative follow-up, he had no complaints and laboratory tests were normal. Abdominal ultrasonography indicated that the hypoechoic region was smaller (Fig. 4). There has been no recurrence.

## Discussion

Lambert reported the first case of liver abscess secondary to gastrointestinal tract perforation by a foreign body in 1898 [6], and other cases have since been reported. Most ingested foreign bodies pass through the gastrointestinal tract uneventfully within a week [7, 8], and only 1% cause perforation. Therefore, a liver abscess caused by an ingested foreign body is extremely rare. Gastrointestinal perforation usually occurs when the foreign body is a fishbone, chicken bone, toothpick, needle, or pen [9]. Perforation can occur at any site in the gastrointestinal tract, and common sites are the pylorus and duodenum [10]. Because of its anatomical location, an abscess caused by a foreign body often involves the left hemiliver [4]. Most patients have no specific symptoms. The classic symptoms of liver abscess (fever with chills, abdominal pain, and jaundice) are uncommon. Most patients have anorexia, vomiting, and weight loss [11]. Actually, our patient’s chief complaints were fever and anorexia. Migration of a foreign body to the liver may be characterized by a long period without symptoms. Therefore, the clinical history is very important. However, most patients do not remember having ingested a foreign body, which makes diagnosis difficult.

Plain radiography may not detect an intra-abdominal abscess, but CT has higher sensitivity and specificity for an abscess or foreign body [12]. Lue et al. reported a study on the ability of plain radiography to detect fish bones in the human body and showed a sensitivity of 39% and specificity of 72% [13]. In another report, CT demonstrated sensitivity as high as 90% [14]. CT should be used for early diagnosis to avoid a fatal liver abscess caused by a foreign body.

The management of a liver abscess caused by a foreign body remains controversial and consists of antibiotic treatment, drainage of the abscess, and removal of the foreign body [15]. Chung et al. [16] suggested that antibiotic monotherapy can be attempted for patients with a pyogenic liver abscess measuring less than 5.0 cm, but recommended percutaneous drainage as the first treatment in patients with an abscess measuring more than 5 cm. On the other hand, Tan et al. [17] reported that surgical drainage provided better clinical postoperative outcomes than percutaneous drainage for liver abscesses measuring more than 5 cm. Only two reported cases of liver abscess caused by a foreign body perforation have been successfully treated conservatively [3, 18]. One case in which percutaneous and surgical drainage were performed without removal of the foreign body resulted in a recurrent liver abscess [15]. Therefore, cases in which liver abscesses develop should undergo removal of the foreign body.

Several reports have described the discovery of a foreign body causing a liver abscess using laparoscopic surgery, as shown in Table 1 [19–25]. In all nine cases, the left liver lobe was involved and the foreign body was present at the liver margins. There were no cases of recurrence. The mean age of these nine patients (five males and four females) was 57 years (range 34–73 years). Drainage and foreign body removal were performed in six of the nine cases. Foreign body removal was performed in all cases. Foreign body removal is essential to prevent recurrence of liver abscess caused by it. As means for removing the foreign body, laparoscopic surgery is very useful in cases in which it is present at the liver margin. Although some cases reported that even a large liver abscess can be treated with minimally invasive laparoscopic surgery after antibiotic therapy and percutaneous drainage, surgery as the first treatment was only used in one case, as in our patient. Regardless of the size of liver abscess, inflammation, and liver enzyme levels, it is very important that early drainage and foreign body removal at the same time without hepatectomy by laparoscopic surgery. The safety of releasing liver abscess in the abdominal cavity is unclear. However, we could prevent forming another abscess for washing the abdominal cavity with a lot of saline and putting drain tube under the liver. It leads to a lot of merits such as no recurrence and reduction of treatment period. Therefore, we chose the surgical treatment as an initial treatment to complete the treatment in a single intervention. Liver abscess cases caused by a foreign body require multidisciplinary treatment, and laparoscopic surgery can be very useful.

**Table 1:** Reports describing the discovery of a foreign body causing a liver abscess using laparoscopic surgery

Case	Year of publication	Author	Patient age	Sex	Abscess position	Maximum abscess size (mm)	Foreign body position	First treatment	Operative method	Recurrence
1	2012	Riani [19]	68	M	Left lobe	Unknown	Margins of the liver	Antibiotics	Left lateral sectionectomy	–
2	2014	Kosar [20]	73	F	Left lobe	60	Margins of the liver	Operation	Foreign body removal + abscess drainage	–
3	2015	Panebianco [21]	57	F	Left lobe	80	Margins of the liver	Antibiotics	Foreign body removal + abscess drainage	–
4	2015	Morelli [22]	65	M	Left lobe	80	Margins of the liver	Antibiotics	Foreign body removal	–
5	2016	Tan [23]	56	M	Left lobe	38	Margins of the liver	Antibiotics + percutaneous drainage	Foreign body removal	–
6	2016	Tan [23]	63	M	Left lobe	90	Margins of the liver	Antibiotics + percutaneous drainage	Foreign body removal	–
7	2018	Bandeira-de-Mello [24]	44	F	Left lobe	95	Margins of the liver	Antibiotics + percutaneous drainage	Foreign body removal	–
8	2018	Yu [25]	34	F	Left lobe	Unknown	Margins of the liver	Antibiotics	Foreign body removal (laparotomy conversion)	–
9	2019	Our case	51	M	Left lobe	55	Margins of the liver	Operation	Foreign body removal + abscess drainage	–

*Abbreviation*: *M* male, *F* female, “–” not detected

## Conclusions

Accurate identification and removal of a foreign body causing a liver abscess is essential. Laparoscopic surgery can be an effective treatment in cases of liver abscess secondary to a foreign body.

## Abbreviations

CRP: C-reactive protein
CT: Computed tomography
WBC: White blood cell
